# The role of complement activation in rhabdomyolysis-induced acute kidney injury

**DOI:** 10.1371/journal.pone.0192361

**Published:** 2018-02-21

**Authors:** XuDong Huang, Wei Zhao, LiXia Zhang, XinJun Yang, LiHui Wang, YunShuang Chen, JingHua Wang, Chao Zhang, GuangLi Wu

**Affiliations:** 1 Department of Nephrology, Chinese PLA General Hospital, Beijing, the People’s Republic of China; 2 Department of Nephrology, Bethune International Peace Hospital, Shijiazhuang, Hebei Province, the People’s Republic of China; Universidade de Sao Paulo, BRAZIL

## Abstract

Rhabdomyolysis (RM) may cause kidney damage and results primarily in acute kidney injury (AKI). Complement is implicated in the pathogenesis of renal diseases and ischemia-reperfusion injury (IRI), but the role of complement, especially its activation pathway(s) and its effect in RM-induced AKI, is not clear. This study established a rat model of AKI induced by RM via intramuscular treatment with glycerol. Cobra venom factor (CVF) was administered via tail vein injection to deplete complement 12 h prior to intramuscular injection of glycerol. We found that the complement components, including complement 3 (C3), C1q, MBL-A, factor B(fB), C5a, C5b-9, and CD59, were significantly increased in rat kidneys after intramuscular glycerol administration. However, the levels of serum BUN and Cr, renal tubular injury scores, and the number of TUNEL-positive cells decreased significantly in the CVF+AKI group. These results suggest that complement plays an important role in RM-induced AKI and that complement depletion may improve renal function and decrease renal tissue damage by reducing the inflammatory response and apoptosis.

## Introduction

Rhabdomyolysis (RM) is characterized by the breakdown of skeletal muscle and the release of intracellular muscle contents into the circulatory system. These cell contents include muscle enzymes, such as creatine phosphokinase (CK), lactate dehydrogenase (LDH), and glutamic oxalacetic transaminase (GOT), the heme pigment myoglobin, electrolytes, such as potassium and phosphate, and purines [1,2]. The spectrum of the syndrome ranges from an asymptomatic elevation of serum muscle enzymes to life-threatening extreme enzyme elevations, electrolyte imbalances, and acute kidney injury (AKI) [3]. AKI is the primary and most severe complication of RM, and approximately half of RM patients present with AKI. Rhabdomyolysis accounts for an estimated 8% to 15% of AKI cases, and it is associated with a mortality rate of 5–8% [4,5]. The mortality rate is highly dependent upon the rapidity of access to medical support, with more prompt treatment corresponding to a lower mortality rate. The mortality rate of rhabdomyolysis induced by earthquakes and other natural disasters is significantly higher than that of rhabdomyolysis that occurs in the hospital due to an inability to be treated quickly.

Different mechanisms are associated with RM-induced AKI, including hypovolemia, intraluminal obstruction by myoglobin, direct myoglobin toxicity, renal ischemia secondary to muscular vasoconstrictors, and free radical production [6–8]. Our recent work demonstrated that the Nrf2/HO-1 pathway plays an important role in an RM-induced AKI rat model [9], but the underlying mechanism is not clear. Several studies in animals have demonstrated that activation of the complement system was a critical cause of AKI and that inhibition of complement activation prevented many of the downstream inflammatory manifestations of AKI [10–12]. The complement system consists of approximately 50 soluble and membrane-bound proteins that are powerful components of innate immunity, and it is highly involved in the inflammatory response [13]. The liver is the primary source of complement production, but increasing evidence from recent studies has demonstrated that many types of resident cells in the kidney produce complement components [14–16].

We hypothesized that inappropriate complement activation is involved in the pathogenesis of RM-induced AKI because of the well-known significance of the complement system in renal injury [10]. We established a rat model of RM-induced AKI through intramuscular glycerol injection into the hind limbs and measured the expression levels of various complement factors: C1q to evaluate the classical pathway; factor B to evaluate the alternative pathway; MBL-A to evaluate the MBL complement pathways; C5a to assess the anaphylatoxic component of the complement system; CD59 to assess complement regulators; and the membrane attack complex. We also evaluated expression changes in inflammation and renal apoptosis in this model.

## Materials and methods

### Animal model and treatment

Male Sprague-Dawley rats weighing 210 ± 10 g were obtained from the Experimental Animal Center of Hebei Medical University (Shijiazhuang, China). The rats were handled and treated in accordance with the guiding principles of the National Institutes of Health for the Experimental Care and Use of Animals, and the Chinese PLA General Hospital Animal Research Ethics Board approved the study. The RM-induced AKI model was generated as previously described [9]. Rats were injected intramuscularly with equal volumes of 10 ml/kg of 50% glycerol into each hind limb. Normal saline was administered instead of 50% glycerol for control rats. Cobra venom factor (CVF) was used as a preventive treatment in this study to deplete complement.

We first demonstrated the ability of CVF to deplete complement and excluded its potential impact on the kidney. Rats were randomly assigned to two groups (24 rats per group): the control group, which received tail vein injection (1 ml/kg) of 10 ml/kg of saline; and the CVF group, which received tail vein injection (50 μg/kg) of CVF (Quidel Corporation, San Diego, CA, USA). All rats received bilateral hind limb muscle injections of 10 ml/kg of saline 12 h later. Six rats were randomly selected from each group and sacrificed via deep anesthesia with pentobarbital (50 mg/kg, i.p.) at 2, 6, 24, and 73 h after injection. Blood samples were collected to test 50% complement hemolysis (CH50) levels and renal function, and kidney tissues were collected for H&E staining to observe morphological changes. After verifying the renal safety of CVF and its efficacy in depleting complement, rats were randomly assigned to three groups (24 rats per group): the control group, which received bilateral hind limb intramuscular injections of 10 ml/kg of saline and tail vein injection (1 ml/kg) of 10 ml/kg of saline 12 h earlier; the AKI group, which received bilateral intramuscular injections of a 50% glycerol saline solution (10 ml/kg) and tail intraperitoneal injection (1 ml/kg) of 10 ml/kg saline 12 h earlier; and the complement-depleted group, in which complement was depleted via tail vein injection (50 μg/kg) of CVF 12 h prior to intramuscular injection of glycerol. No rats died before the samples were collected. Six rats were randomly selected from each group and sacrificed via deep anesthesia with pentobarbital (50 mg/kg, i.p.) at 2, 6, 24, and 72 h after glycerol injection. Venous blood and renal tissue samples were then collected.

### Serum analysis

Renal function and C-reactive protein (CRP) levels were analyzed using a Hitachi 7170 automated biochemistry analyzer (Tokyo, Japan). Rat CH50 levels in serum were measured using ELISA kits (MyBioSource, MA, USA) following the manufacturer’s instructions. Optical density was measured using a microplate reader (Infinite F50, TECAN, Salzburg, Austria) at 450 nm. A standard curve was drawn using Magellan software (TECAN), which calculated the CH50 level in each sample.

### Renal morphology

Kidneys were removed, and sagittal sections were fixed in a 4% paraformaldehyde solution overnight, embedded in wax, and sectioned at a thickness of 4 μm. Kidney tissues were stained with hematoxylin-eosin (H&E) and periodic acid-Schiff (PAS). Tubular injuries were evaluated blindly and scored semi-quantitatively using a previously reported scoring system [17]. Tubular injury in the outer medulla and corticomedullary junction was defined by tubule dilation, necrotic lysis, cast formation, sloughing of cellular debris into the tubule lumen, or the presence of a naked tubule basement membrane. Tubular injury was scored as follows: 0, no injury; 1, <20%; 2, 21–50%; 3, >50%; and 4, total destruction of all epithelial cells.

### Immunofluorescence

Cryostat sections (4-μm) of frozen kidneys were stained for complement 3 (C3), C1q, and complement factors B (fB), MBL-A, C5a, C5b-9, and CD59. Slides were air-dried, fixed with methanol/acetone for 10 min, and treated with the following FITC-conjugated anti-rat antibodies (Abs): rabbit anti-rat C3 Ab (Abcam, Cambridge, MA, USA); rabbit anti-rat C1q Ab (Abcam); rabbit anti-rat MBL-A Ab (Abcam); rabbit anti-rat factors B Ab (Santa Cruz, USA); rabbit anti-rat C5a Ab (ABBIOTEC, USA); and rabbit anti-rat CD59 Ab (Santa Cruz). At least 10 high-powered fields in the outer medulla and corticomedullary junction were assessed and scored as follows [18]: Grade 0, negative; Grade 1, <25% positive staining; Grade 2, <50%; Grade 3, <75%; or Grade 4, >75%.

### Immunohistochemistry

Four-micrometer-thick sections were incubated with 0.3% H_2_O_2_ for 20 min and 10% goat serum for 1 h to reduce non-specific background staining. The sections were incubated in a 100°C water bath for 10 min and blocked with 2% BSA for 30 min. The sections were then incubated with the following anti-rat antibodies overnight at 4°C: anti-rat IL-6 Ab (1:100, Santa Cruz) and anti-rat CD68 Ab (1:100, Santa Cruz). At least 5 high-powered fields in the outer medulla and corticomedullary junction were observed and semi-quantified using Image-Pro Plus 6.0 software (Media Cybernetics, USA).

### Western blot analysis

Briefly, tissues between the renal cortex and medulla were homogenized with RIPA lysis buffer on ice and centrifuged at 12,000 rpm and 4°C for 10 min. Protein concentrations for all samples were determined using a BCA Protein Assay Kit (Bioword Technology, St. Louis Park, USA). Protein (50 μg) from each sample was resolved using electrophoresis with a 10% Bis-Tris polyacrylamide gel (Millipore, Billerica, USA) and transferred to a nitrocellulose membrane. Membranes were blocked with 5% fat-free milk in TBST buffer and incubated with polyclonal anti-rat C1q Ab (1:1,000, Abcam), MBL-A Ab (1:500, Abcam), f B Ab (1:500, Santa Cruz), C5a Ab (1:500, ABBIOTEC, USA), CD59 Ab, IL-6 Ab (1:1000, Santa Cruz), and GAPDH (1:5,000, Bioword Technology, St. Louis Park, USA) at 4°C overnight. The results were densitometrically quantified using Image-Pro Plus 6.0 software.

### Real-time qPCR

Kidney tissues between the renal cortex and medulla were obtained from rats at the indicated time points. Total RNA was extracted using TRIZOL (TianGen Biotech, Beijing, China) and reverse transcribed into cDNA using a ReverTra Ace qPCR RT kit with a gDNA Eraser (TaKaRa Biotechnology, Otsu Shiga, Japan). The cDNA was amplified using PCR. Quantitative PCR with SYBR Green Real-time PCR Master Mix-Plus (Toyobo) was performed using a Prism 7500 Sequence Detection System (Life Technologies, CA, USA). The mRNA levels were normalized to the housekeeping gene GAPDH. The following DNA sequences were used for the primer pairs: C3 forward 5- GCATCAGTCACAGGATCAGGTCA -3’ and C3 reverse 5’- ATCAAAATCATCCGACAGCTCTATC -3’; C1q forward 5- GATGGCTGGTGGCTTGTGT-3’ and C1q reverse 5’- CAGATTCCCCCATGTCTCCTT -3’; MBL-A forward 5- CTATGCCGAGACCTTAACCGA -3’ and MBL-A reverse 5’- CTTTTGGTCCCGTTGCTCC -3’; fB forward 5- AGAAAGGCGGTGATTATTACAAGC -3’ and fB reverse 5’- GGTGGAACAGGACCTCTTCAATC -3’; IL-6 forward 5- GATTGTATGAACAGCGATGATGC -3’ and IL-6 reverse 5’- AGAAACGGAACTCCAGAAGACC -3’; GAPDH forward 5- TGGAGTCTACTGGCGTCTT -3’ and GAPDH reverse 5’- TGTCATATTTCTCGTGGTTCA -3’. Quantitative PCR assays were conducted in triplicate, and quantification was performed using the F = 2^−ΔΔct^ method.

### Assessment of apoptosis using TUNEL staining

TUNEL staining was performed using an apoptosis detection kit (Roche Diagnostics, GmbH- Mannheim, Germany) according to the manufacturer’s instructions. Rat spleen sections were used as positive controls. Two blinded observers independently analyzed the results using Image-Pro Plus 6.0 software.

### Statistical analysis

All statistical analyses were performed using SPSS 17.0 for Windows. The data are expressed as the means ± standard error of the mean. P<0.05 was accepted as statistically significant. Renal function, renal complement protein expression levels, and renal mRNA expression levels were compared between the treatment arms via one-way ANOVA. The S-N-K test was used for post hoc multiple comparisons.

## Results

### CVF can efficiently deplete complement and has no effect on the rat kidneys

**Fig 1:**
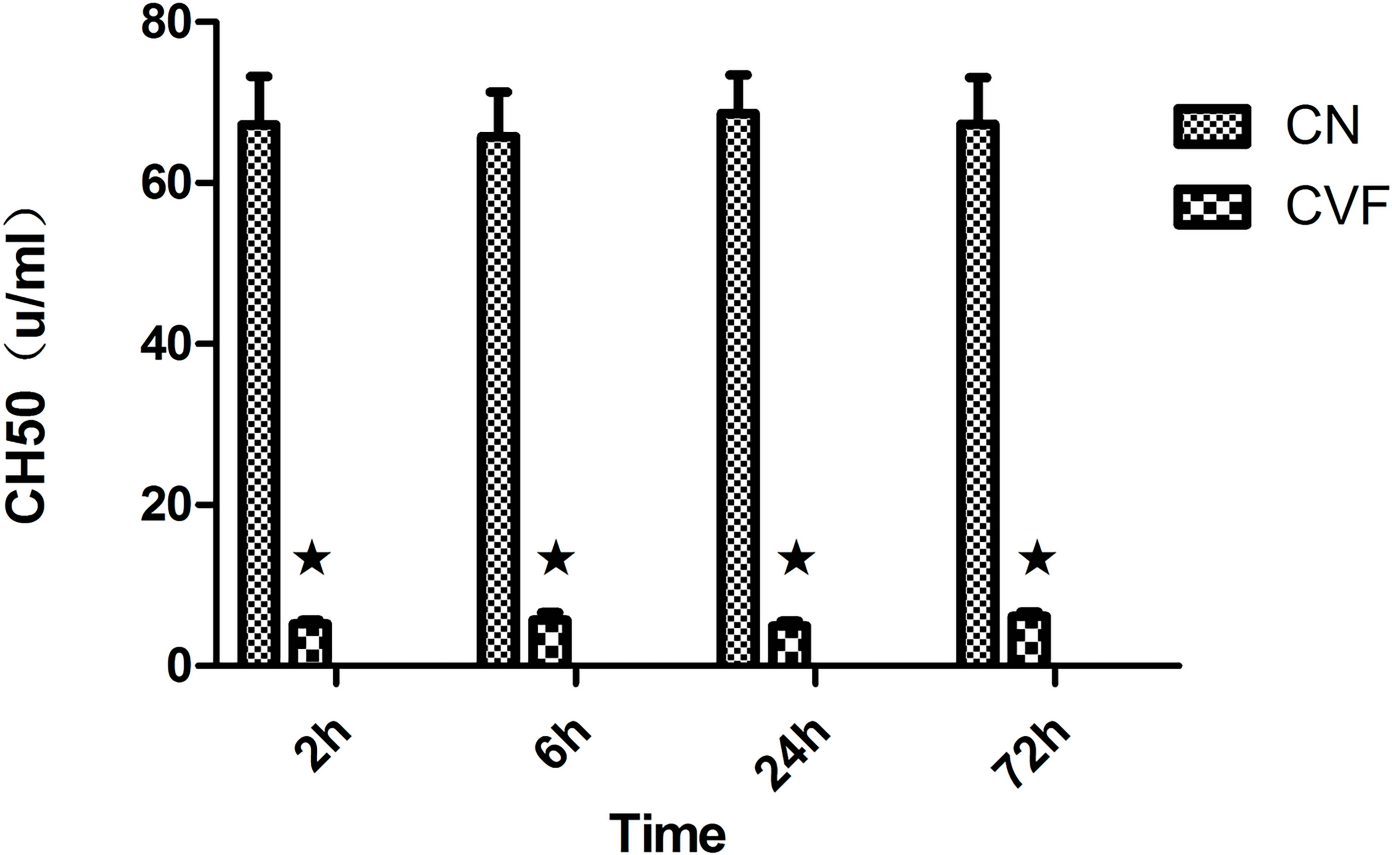
Levels of serum CH50. Each bar represents the mean±SD. ★ P<0.01 vs. control.

CH50 represents total complement activity. To detect the ability of CVF to deplete complement, we detected the CH50 in rat serum. Rat serum CH50 levels decreased significantly to less than 10% of the levels in the control group after intravenous CVF administration and remained at a very low level for a long time (Table 1, Fig 1). The results indicated that CVF depleted rat serum complement.

**Table 1:** Levels of serum CH50 in the two groups (u/ml, means±SD, n = 6).

Group	2 h	6 h	24 h	72 h
**Control**	67.24±6.02	65.74±5.58	68.62±4.84	67.32±5.81
**CVF**	5.24±0.49*	5.69±0.92*	4.94±0.66*	6.17±0.52*

* P<0.01 vs. control

**Fig 2:**
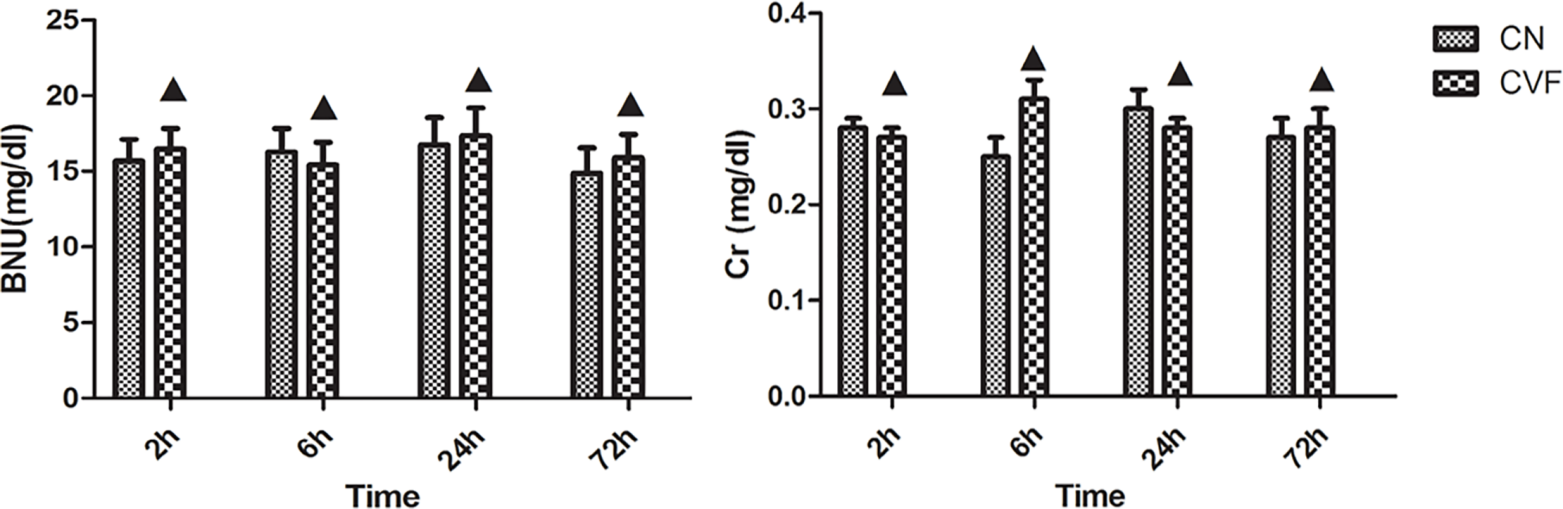
Levels of Serum BUN and Cr. ▲ P>0.05 vs. control.

**Fig 3:**
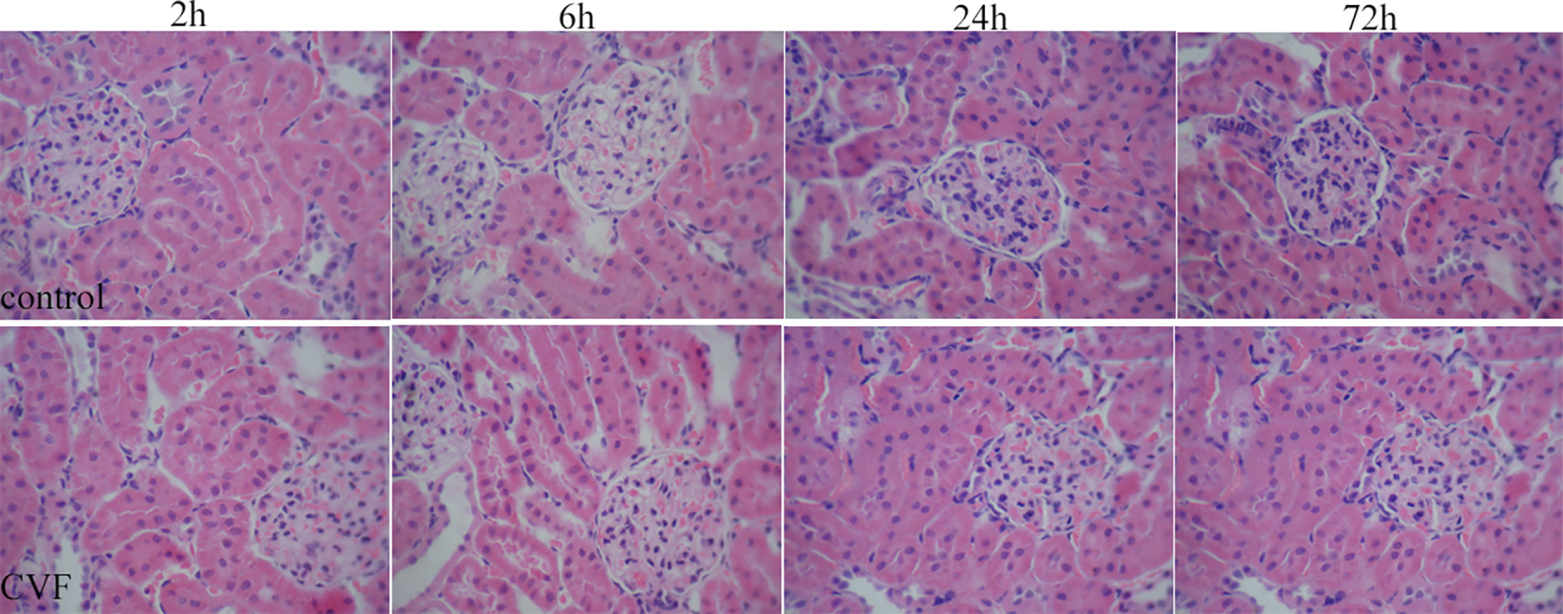
Evaluation of renal histopathology changes. No change in renal morphology was observed at four time points in the CVF group compared with that in the control group.

To verify the renal safety of CVF, we tested rat serum creatinine (Cr) and urea nitrogen (BUN) levels, and the results showed that the renal function of the rats did not change during the observation period after CVF administration (Fig 2& Table A in S1 File). Renal H&E staining also revealed that the CVF group’s kidney structure did not change over time (Fig 3). These results indicate that CVF had no effect on the kidneys of rats.

### The complement system may influence RM-induced AKI

**Fig 4:**
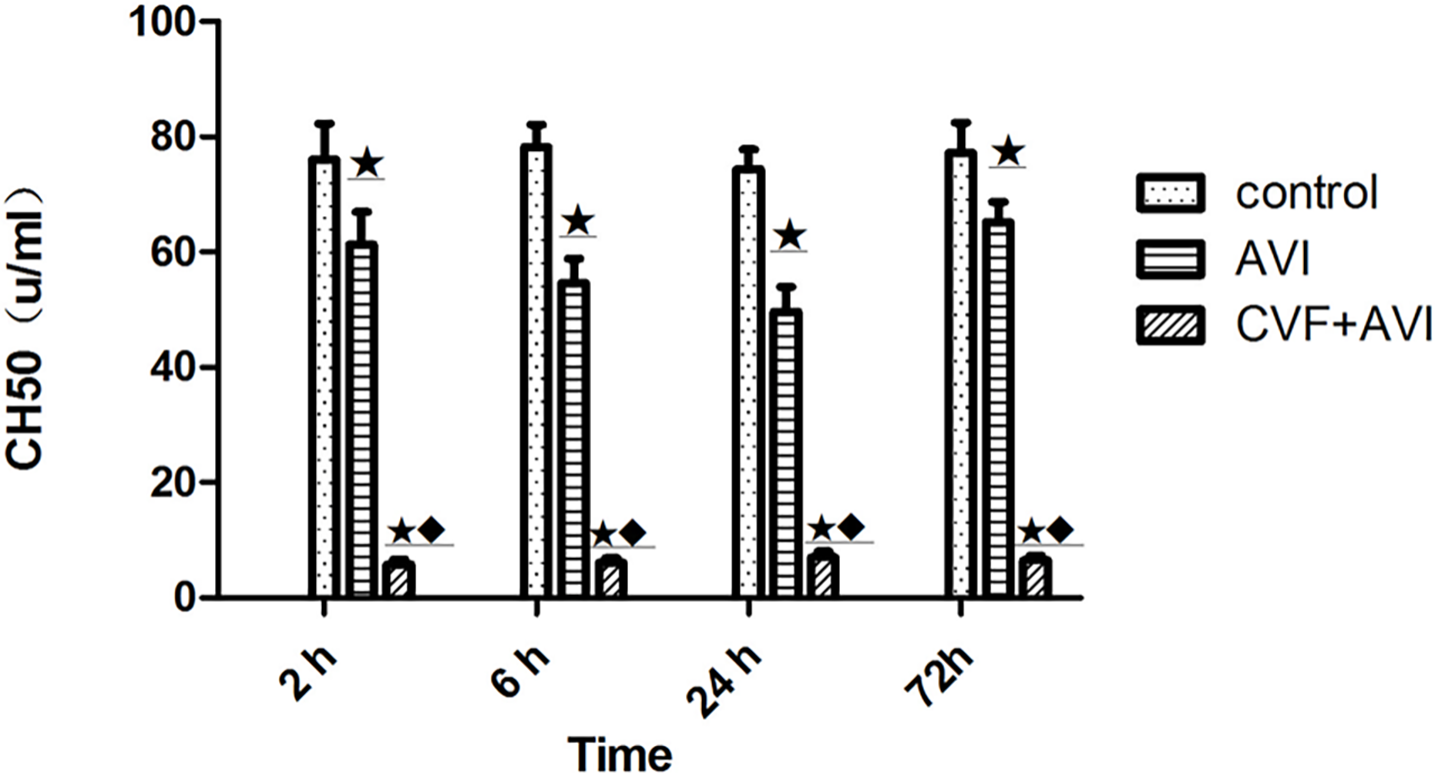
Levels of serum CH50. ★ P<0.01 vs. control; ◆ P<0.01 vs. AKI.

Rat serum CH50 levels in the CVF+AKI group decreased significantly compared with those in the control group and the AKI group (Table 2, Fig 4). The results reaffirmed that CVF depleted rat serum complement.

**Table 2:** Levels of serum CH50 in the three groups (u/ml, means±SD, n = 6).

Group	2 h	6 h	24 h	72 h
**Control**	76.05±6.28	78.24±3.87	74.35±3.41	77.24±5.21
**AKI**	61.28±5.64*	54.64±4.26*	49.64±4.29*	65.21±3.46*
**CVF+AKI**	5.75±0.38*#	6.12±0.74*#	7.03±1.04*#	6.55±0.78*#

* P<0.01 vs. control

# P<0.01 vs. AKI

**Fig 5:**
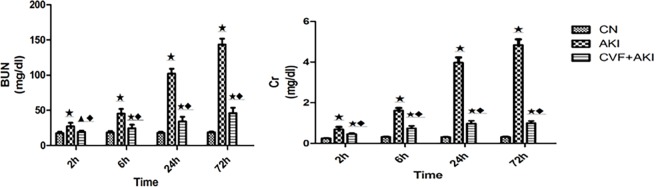
Levels of serum BUN and Cr. ★ P<0.01▲ P>0.05 vs. control; ◆ P<0.01 vs. AKI.

We used CVF to deplete rat serum complement to investigate the role of complement in this model by observing renal function and morphological changes. After intramuscular injection of glycerol, rat serum markers of renal function increased over time. If CVF was given prior to the injection of glycerol, the increase in the renal function index was significantly diminished. BUN levels were decreased from 143.55±8.30 mg/dl at 72 h in the AKI group to 46.16±7.6 mg/dl at the same time point in the CVF+AKI group, and Cr levels decreased from 4.84±0.18 mg/dl to 0.99±0.11 mg/dl. The results demonstrated that complement depletion markedly improved kidney function. (Table 3, Fig 5).

**Table 3:** Levels of serum BUN and Cr in the three groups (means±SD, n = 6).

Group	Time	BUN (mg/dl)	Cr (mg/dl)
**Control**	2 h	17.72±1.85	0.25±0.02
	6 h	18.43±1.98	0.32±0.02
	24 h	17.95±1.59	0.31±0.02
	72 h	18.57±1.34	0.32±0.02
**AKI**	2 h	27.19±5.09*	0.69±0.09*
	6 h	45.23±6.75*	1.61±0.09*
	24 h	102.02±7.12*	3.97±0.16*
	72 h	143.55±8.30*	4.84±0.18*
**CVF+AKI**	2 h	19.38±1.75^●^^#^	0.46±0.05*^#^
	6 h	24.43±5.15*^#^	0.74±0.12*^#^
	24 h	34.23±6.54*^#^	0.97±0.14*^#^
	72 h	46.16±7.6*^#^	0.99±0.11*^#^

Abbreviations: BUN, urea nitrogen; Cr, creatinine.

* P<0.01

^●^ P>0.05 vs. control

# P<0.01 vs. AKI

**Fig 6:**
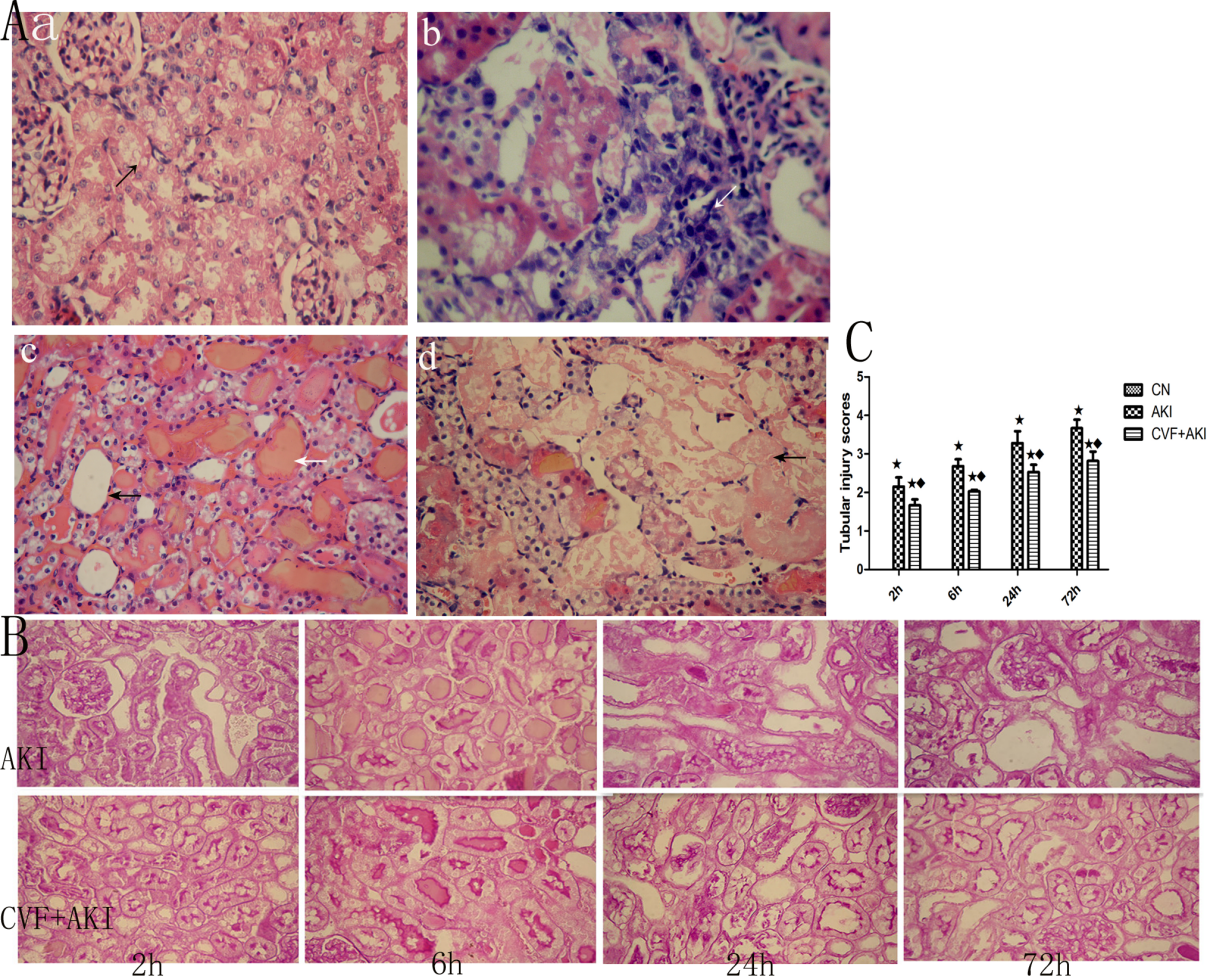
Evaluation of renal histopathology changes. (A) Renal injury in the AKI group was aggravated over time. a. AKI at 2 h, renal tubular epithelial cell degeneration (H&E, ×200); b. AKI at 6 h, inflammatory cell infiltration (H&E, ×400); c. AKI at 24 h, the black arrow indicates the bare basement membrane, and the white arrow indicates the myoglobin tube type (H&E, ×200); d. AKI at 72 h, large patchy necrosis, normal structures disappear (H&E, ×200). (B) The pathological renal changes in the AKI+CVF group were more moderate than those in the AKI group (PAS, ×200). (C) Tubular injury scores of each group. The score of the control group was zero. ★ P<0.01 vs. control; ◆ P<0.01 vs. AKI.

We previously demonstrated that RM induced injury based on renal morphology [9]. Renal H&E and PAS staining revealed that the AKI group exhibited an obvious and progressive injury pattern over time. Mild lesions were observed at 2 h, primarily degeneration of tubular epithelial cells, renal tubular epithelial cells exhibited granular and vacuolar degeneration, and homogeneous red-staining protein casts appeared in individual renal lumens of the outer medulla and corticomedullary junction. Large cell debris in the renal tubules and lumen, multiple protein casts in the renal lumen, tubular dilatation, and inflammatory cell infiltration appeared at 6 h. Renal tubules lost their normal structure, and epithelial cell necrosis, a bare basement membrane, dissolution, and epithelial cell shedding were observed at 24 h. More renal tubular epithelial cell shedding, necrotic cell debris in the lumen, and cell tube types were observed at 72 h, but partial repair phenomena and naked basement membrane rearrangement also appeared (Fig 6A). Depletion of complement ameliorated rat kidney tissue damage (Fig 6B) and lowered tubular injury scores (Fig 6C).

**Fig 7:**
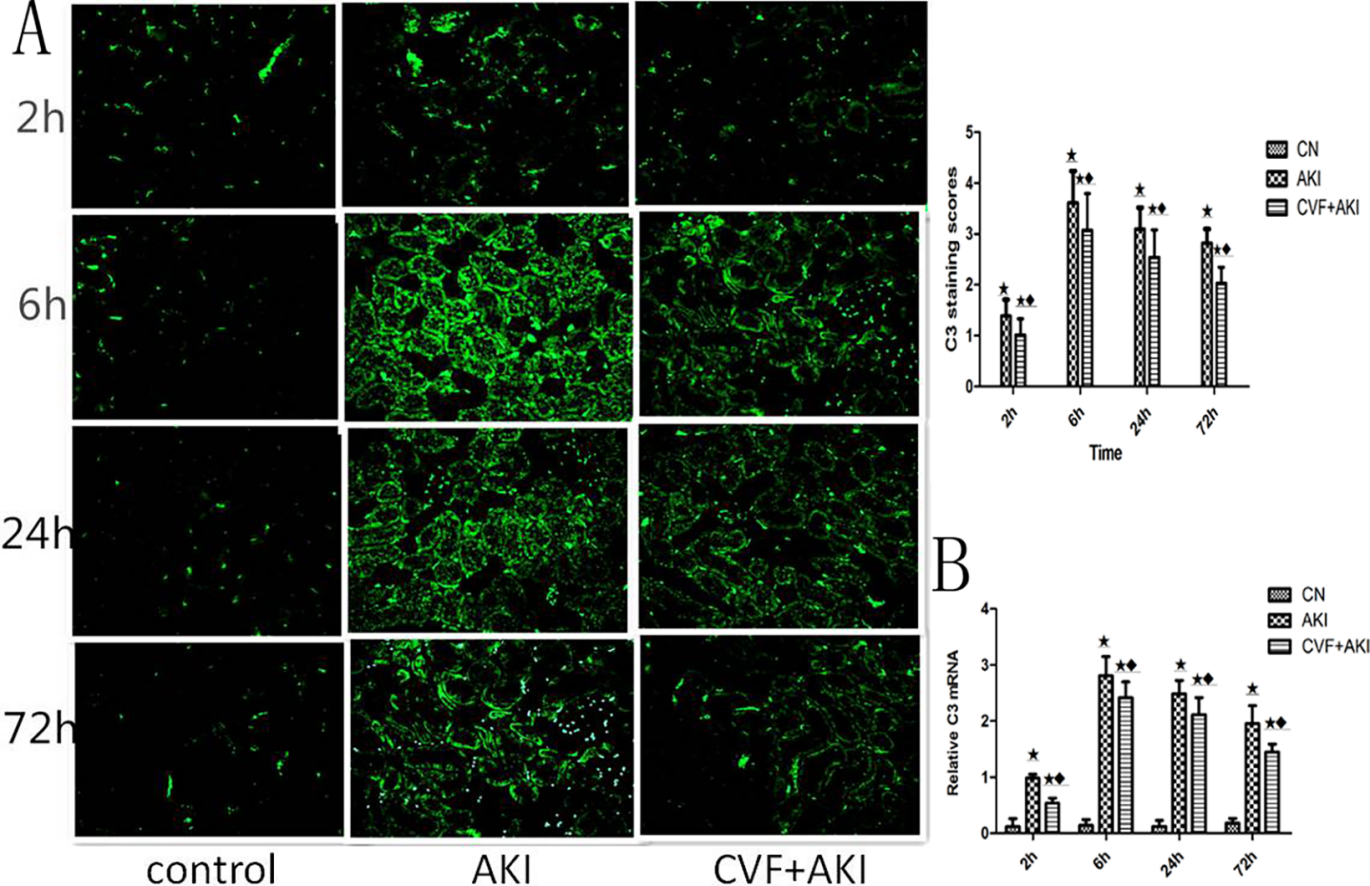
Evaluation of renal C3 changes. (A) Immunofluorescence staining for C3. C3 was deposited along the renal tubular basement membrane in the experimental groups, but not in the control group. (B) C3 mRNA expression levels in the different groups. ★ P<0.01 and ▲ P>0.05 vs. control; ◆ P<0.01 vs. AKI.

These results demonstrated that complement was involved in RM-induced AKI, and complement depletion significantly reduced renal damage in this model. We also examined renal C3 to confirm these observations. C3 is the most abundant complement protein, and its content increases after complement activation. Immunofluorescence demonstrated that kidneys in the control group did not express C3, but C3 expression in the AKI group was significantly enhanced. Fluorescence was positive at 2 h, primarily along the tubular basement membrane in the outer medulla and corticomedullary junction, and it was stronger at 6 h. The signal strength weakened, but it was still significantly stronger than that in the control group at the subsequent time points. Real-time qPCR revealed reduced C3 mRNA in the renal tissues of complement-depleted rats (Fig 7).

### Complement may be activated via three pathways in RM-induced AKI

Complement normally circulates in the peripheral blood as an inactive zymogen that may be activated by exogenous or endogenous stimuli to initiate a cascade reaction. Complement plays a role only when activated. Complement activation produces proteolytic cleavages, structural rearrangements, and lytic complexes. Complement is omnipresent in an inactive form but becomes activated locally. There are three ways to activate the complement pathway: the classical pathway (CP), the alternative pathway (AP), and the lectin pathway (LP). We also investigated the possible activation routes of complement in this model.

**Fig 8:**
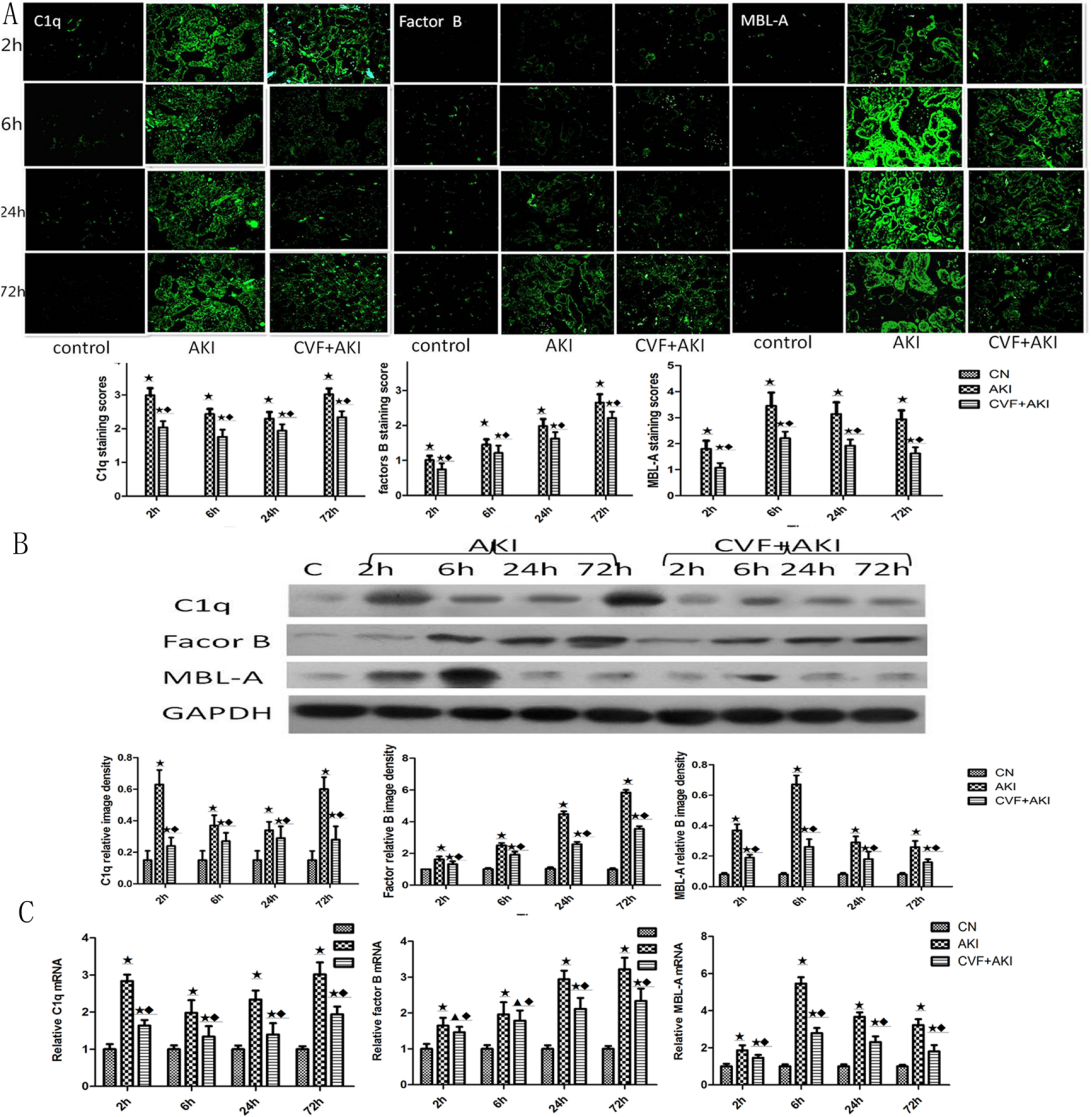
Evaluation of C1q, fB, and MBL-A changes in kidneys. (A) Immunofluorescence staining for three complement activator-associated proteins. (B) C1q, fB, and MBL-A protein levels were measured using western blot analysis; the ratio of complement expression to that of GAPDH was determined using densitometry (n = 6). (C) The mRNA expression levels of these proteins in the different groups. Protein and RNA levels in the kidneys of AKI rats were higher than those in the control group, but the amplitude of this increase was lower after the depletion of complement. ★ P<0.01, ▲ P>0.05 vs. control; ◆ P<0.01 vs. AKI.

C1q is the initiating protein of the CP. Positive C1q staining was observed along the tubular basement membrane 2 h after intramuscular glycerol injection. Fluorescence intensity decreased at 6 and 24 h, but it increased again at 72 h. fB is an essential component of the AP. We used western blotting to analyze fB protein in rat kidneys. The results demonstrated a progressive elevation in fB levels after intramuscular glycerol injection. We also detected MBL-A mRNA in rat kidneys. MBL-A mRNA levels increased 2 h after intramuscular glycerol injection and peaked at 6 h (Fig 8 & Figures A-C in S2 File).

### How does activated complement affect RM-induced AKI?

**Fig 9:**
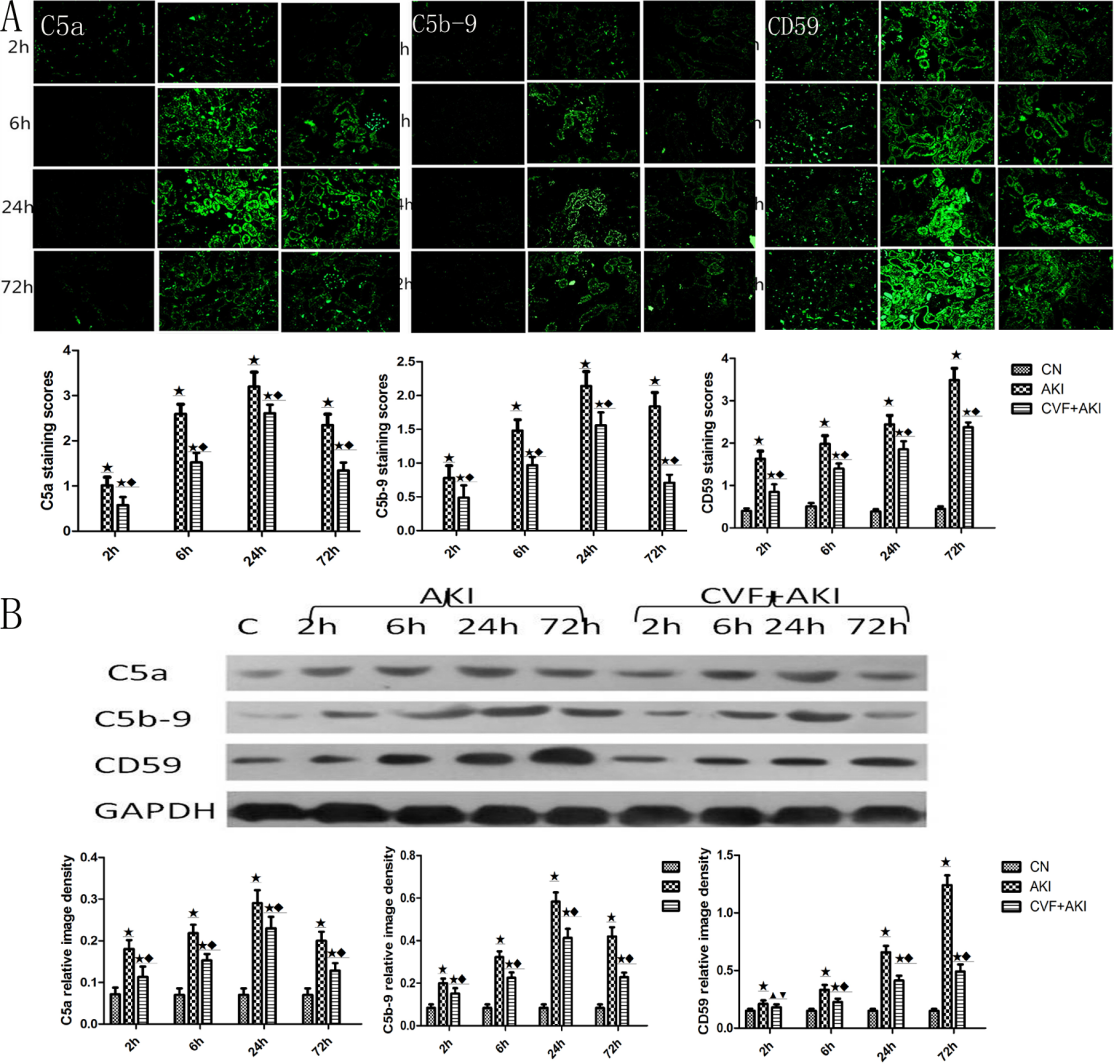
Evaluation of C5a, C5b-9, and CD59 changes in the kidney. (A) Immunofluorescence staining of three complement proteins. (B) C5a, C5b-9, and CD59 protein levels were measured using western blot analysis, and the ratio of these proteins to that of GAPDH was determined using densitometry. ★ P<0.01 and ▲ P>0.05 vs. control; ◆ P<0.01 and ▼ P>0.05 vs. AKI.

We measured renal C5a, C5b-9, and CD59 to examine the roles of complement components in RM-induced AKI. The results revealed no C5a or C5b-9 fluorescence and weak CD59 fluorescence in the control group. However, fluorescence increased significantly in the renal tubules, primarily along the tubular basement membrane, after intramuscular glycerol injection. The strongest fluorescence intensities of C5a and C5b-9 were observed at 24 h, but the strongest CD59 fluorescence was observed at 72 h. Western blotting revealed that renal protein levels decreased significantly in the complement-depleted rats compared with those in the AKI rats at all time points (Fig 9).

**Fig 10:**
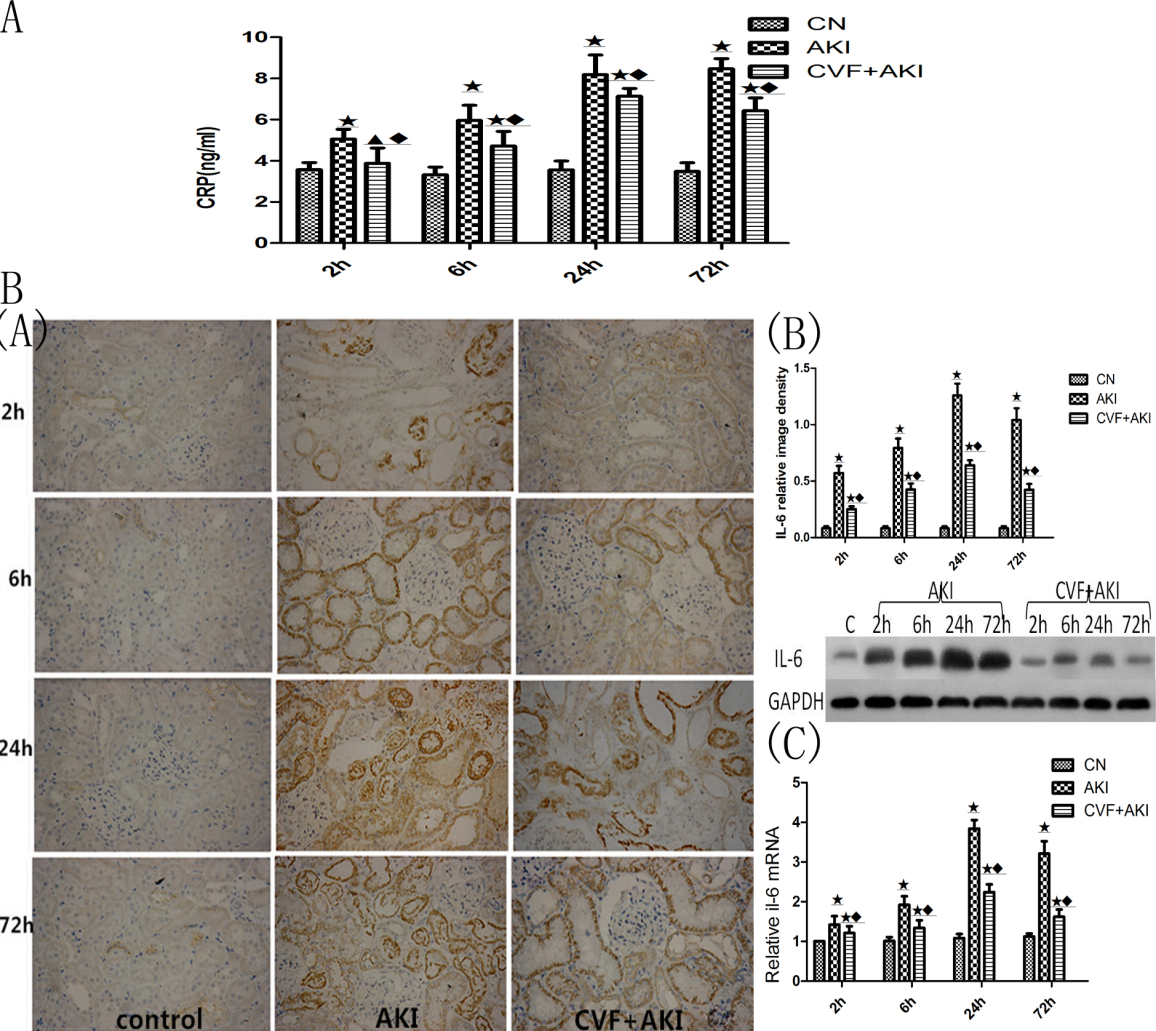
Evaluation of CRP and IL-6 changes in the kidney. A. CRP levels in serum. B. (A) immunohistochemical staining for IL-6 (×400). (B) IL-6 protein levels obtained by western blot analysis. (C) mRNA expression levels of IL-6 in rat kidney tissues. ★ P<0.01 vs. control; ◆ P<0.01 vs. AKI.

**Fig 11:**
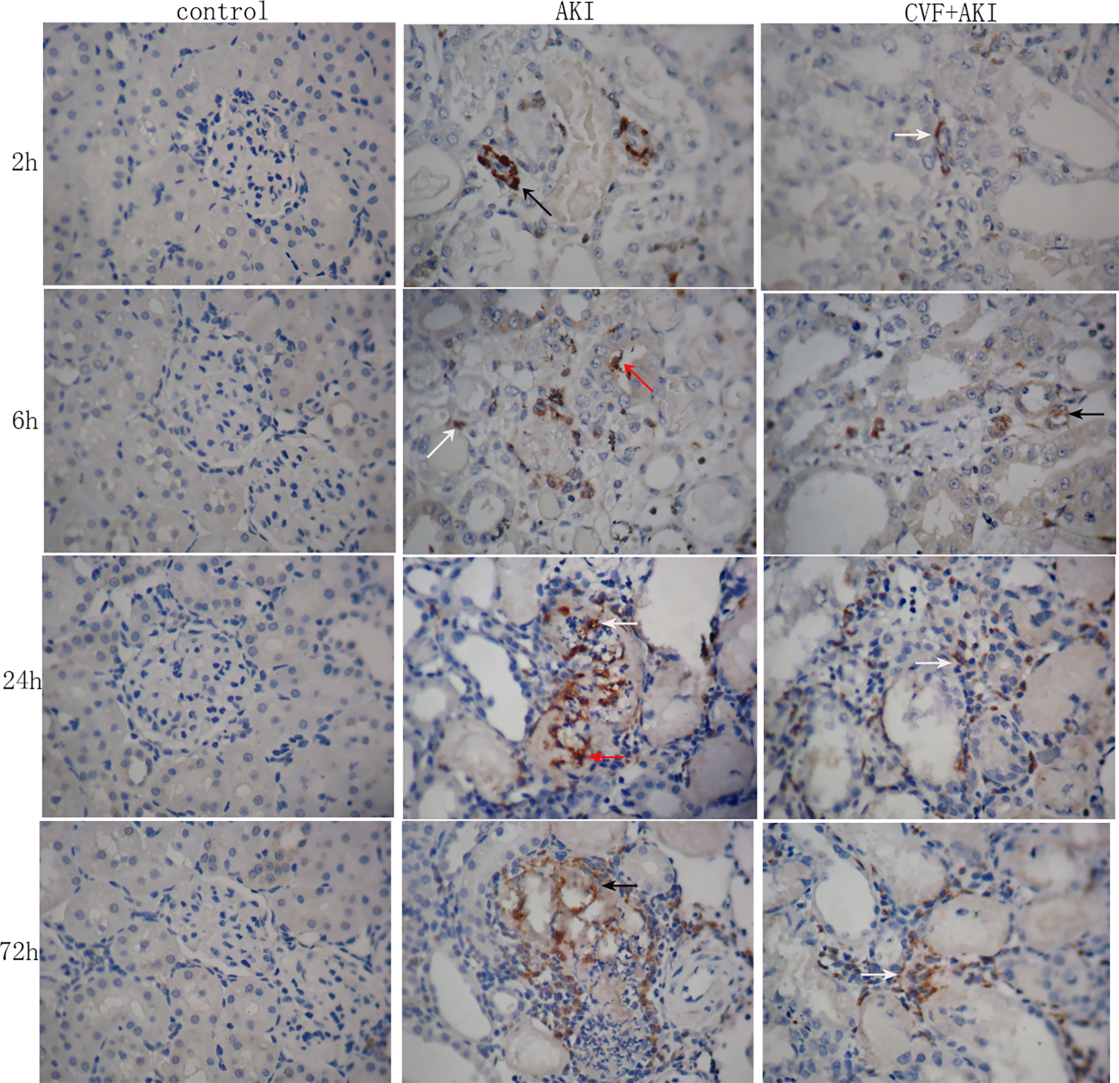
Immunohistochemical staining for CD68. CD68^**+**^ macrophages are located in the renal interstitium indicated by white arrow, blood vessels indicated by black arrow or the lumen of the renal tubules indicated by red arrow (×400).

Complement is a major component of the body's immune defense, and it significantly influences the inflammatory response. We examined inflammatory-related factors, including CRP levels in serum and CD68 and IL-6 expression in kidneys, to elucidate the effects of complement on the inflammatory response. The serum CRP concentration in the AKI group increased significantly compared with that in the control group at the first time point (P<0.01) and then increased gradually (P<0.01). The serum CRP concentration in the CVF+AKI group also increased significantly compared with that in the control group, except at the first time point, but the concentration decreased significantly compared with that in the AKI group at every time point. We used immunohistochemistry to observe CD68 and IL-6 expression in the kidneys, and the results demonstrated an obvious increase after glycerol injection, primarily in the renal interstitium, blood vessel or the lumen of the renal tubules for CD68 and in the cytoplasm of renal tubular epithelial cells for IL-6, which were stained with yellowish-brown granules. All expression was greatest at 24 h and weakened with the depletion of complement prior to glycerol injection (Figs 10 and 11 & Figures D and E in S2 File).

**Fig 12:**
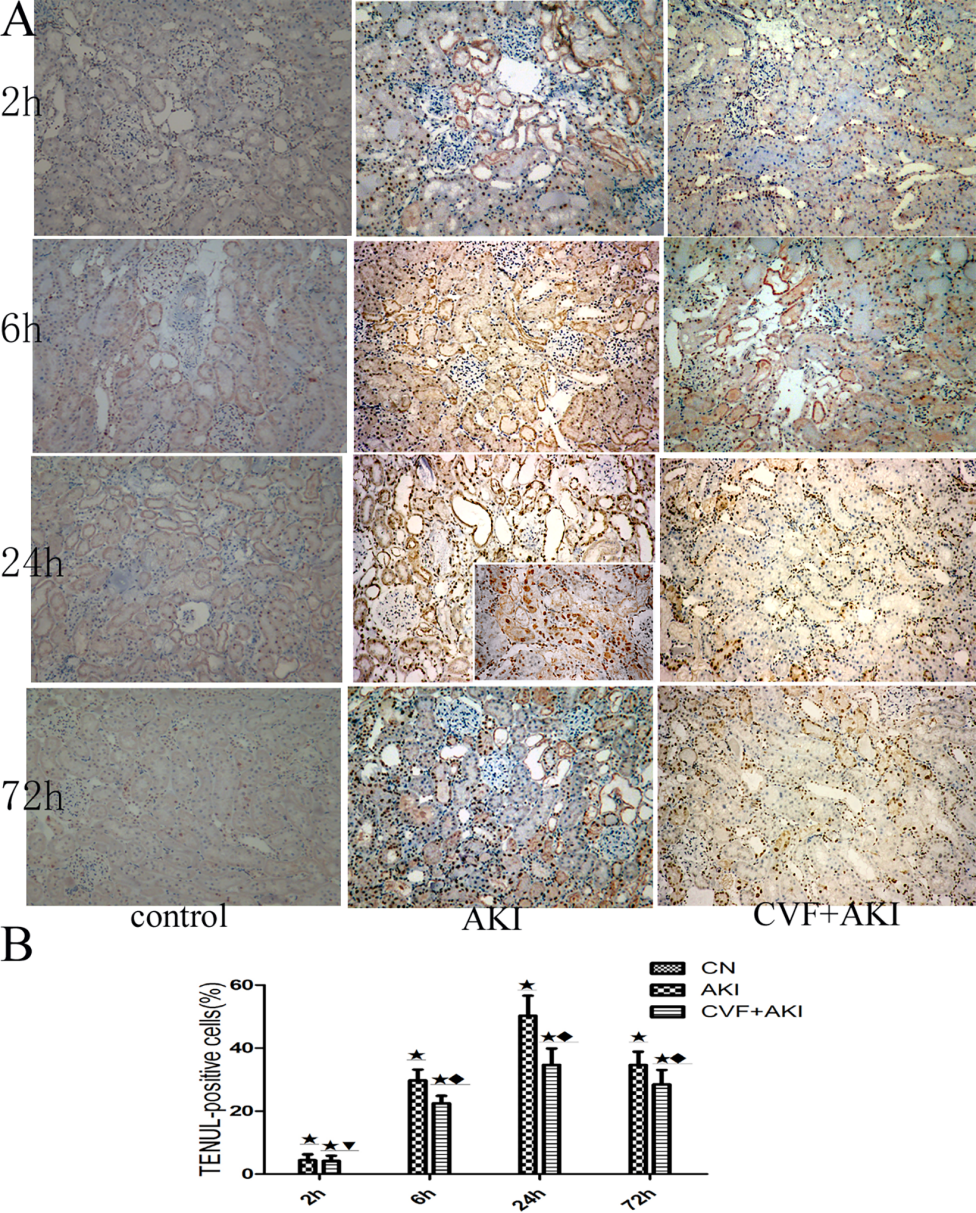
TUNEL assessment of renal tubular epithelial cell apoptosis. (A) Renal tissue sections underwent TUNEL staining (200× magnification). The nuclei of TUNEL-positive cells are stained brown. (B) The level of apoptosis is indicated as the percentage of TUNEL-positive cells with respect to the total number of cells.

Complement activation also triggers apoptosis signaling. Previous studies using a brain ischemia-reperfusion (I/R) model found increased expression levels of complement, and NF-κB was associated with enhanced cerebral apoptosis [19,20]. We used the TUNEL assay to evaluate the level of renal tubular epithelial cell apoptosis and to further investigate the role of complement in RM-induced AKI. Positively stained nuclei were occasionally observed in the renal tissues of the control group. TUNEL-positive cells increased significantly after glycerol injection, primarily in the renal tubules of the outer medulla and corticomedullary junction. Half of the cell nuclei were strongly stained at 24 h in the AKI group. The number of apoptotic cells in renal tissues also increased in the complement-depleted group, but this number was significantly decreased compared with that in the AKI group (Fig 12 & Table B in S1 File).

## Discussion

Intramuscular injection of glycerol efficiently reproduces the clinical syndrome of RM-induced AKI in humans. Our previous research revealed that serum myoglobin was obviously increased during the very early stages of renal injury [9], and the results of the present study revealed associated renal dysfunction. BUN and Cr levels increased steadily and significantly, and renal histology revealed damaged tissue. The normal structure at the junction of the cortex and medulla disappeared at 72 h. RM-induced AKI is a complex process, and the exact pathological mechanism is not clear. Research has demonstrated that complement plays a pathological role in a variety of renal diseases [21–24]. For example, uncontrolled AP activation within the kidney is the primary cause of scleroderma renal crisis (SRC) [25]. The complement system is also the primary cause of atypical hemolytic uremic syndrome (aHUS), with uncontrolled activation within the microvasculature [26]. Complement is activated via the AP during the early phase of reperfusion in renal IRI. These results suggest that the kidney may be intrinsically susceptible to complement-mediated injury.

We used CVF to deplete complement prior to intramuscular glycerol injection to elucidate the role of complement. CVF is a nontoxic protein found in cobra venom that forms an inactivator-resistant convertase of C3/C5 and continuously activates C3 and C5. The complement elimination function of CVF is efficient and long-lasting. Only a single injection of CVF at 6.8 nmol/kg reduced the complement content in mouse serum to trace amounts, and this effect lasted for 2–4 days [27]. Our results also showed that after intravenous CVF administration, rat serum complement levels were very low within 84h. Other complement antagonists include sCR1, C1 Inhibitor, and eculizumab. Eculizumab, a monoclonal anti-C5 antibody, has been reported to be effective in controlling thrombotic microangiopathy and improving renal function, and is used in clinical practice to treat paroxysmal nocturnal hemoglobinuria (PNH) and atypical hemolytic-uremic syndrome (aHUS). Eculizumab as a C5 blocker is a terminal cascade blocking agent, and C3 convertase initially remains active. Therefore, we chose CVF as a tool to deplete complement. Our results confirm that there was no change in renal function or morphology after intravenous CVF injection in rats. Because CVF depletion of complement is efficient and safe, CVF has been used as a tool to study the biological functions of complement and the pathogenesis of some diseases [28]. CVF pre-treatment in the RM-induced AKI model improved renal function and significantly reduced serum BUN and Cr levels and renal tissue damage. These results indicate that complement plays a key role in RM-induced AKI.

C3 is the most abundant complement protein in the body, and it exhibits a certain degree of complement activation. C3 content is increased significantly in some cardiovascular diseases [29], and cleavage of C3 is the main step in the complement activation cascade. In the RM rat kidney, the expression of C3 along the renal tubular basement membrane increased significantly, and this site of expression is consistent with tissue injury. Renal C3 expression increased despite CVF-induced depletion of complement in the peripheral blood. These results suggest that renal tissue synthesizes C3 and that the complement system is activated in RM. The expression level of C3 protein peaked 6 h after glycerol treatment and then decreased to a level that remained significantly higher than normal, suggesting that C3 decomposition was faster than C3 synthesis.

The AP is the major, or only, route of complement activation in kidney disease. In a model of acute renal injury induced by ischemia-reperfusion and sepsis in mice, decreased or defective fB or fP activity can reduce renal injury and inflammatory responses [30–32]. Explanations for this observation include the following: the immunoglobulin and complement proteins in plasma cannot pass through the glomerular filtration membrane; the ammonia synthesized by the renal tubule promotes AP activation; and the acidic environment of the damaged renal tissue is conducive to AP activation [33,34]. This study investigated the complement activation pathway in RM-induced AKI. Our data demonstrated that the expression of fB, as a representative of the AP, was elevated in the kidney after intramuscular glycerol injection. The progressive increase in fB is also related to the release of cellular microparticles by necrotic cells due to the aggravation of kidney injury, which could also induce AP activation [35]. In the CP and LP approaches to acute kidney damage, the results from different labs are inconsistent. Some studies suggest that the CP does not participate in acute kidney damage, and in a renal ischemia-reperfusion model, C1q deposits were not found in renal tissues [30]. One study found that MBL gene knockout mice exhibited decreased plasma C3a levels and reduced kidney damage after renal ischemia-reperfusion injury, and the injury could be recovered by injection of recombinant MBL [33].Our data demonstrated elevated C1q and MBL-A expression in the kidneys of RM rats. C1q is an initiating protein of the CP. The structure of MBL-A is highly homologous to that of C1q, and it is one of two different forms of MBL (MBL-A and MBL-C) in rats. Rat MBL-A is dominant and exhibits 66.7% homology with human MBL. Elevation of these factors suggests that the complement system was activated via the CP and LP in RM-induced AKI. We previously found that reperfusion injury of ischemic skeletal muscle invoked complement activation primarily via the CP and LP [36–38]. Earlier injury to skeletal muscle in our model was the predisposing factor for kidney injury. Elevated C1q and MBL-A during early kidney injury may be related to complement activation in skeletal muscle. Complement was activated in skeletal muscle during RM via the CP and/or LP, and some activated complement proteins reached the kidney via blood circulation and were deposited in the kidneys. These complement proteins activated the renal complement pathway via the CP and/or LP. C1q also initiates the complement activation program in conjunction with CRP and apoptotic bodies [39–41]. Our experimental results demonstrated that serum CRP levels and the apoptotic rate in renal tissues gradually increased after glycerol injection and peaked in the late stage of injury. The second increase in C1q content that occurred at 72 h may be related to this finding.

Complement is the most important immune defense system in the body. Complement defends against pathogenic microorganisms, maintains homeostasis, and prevents autoimmunity. Complement also has an obvious promoting effect in aseptic inflammation. Serum CRP concentrations increased significantly in RM, and this elevation decreased significantly after CVF treatment. These results suggest that the increase in serum CRP was partially related to complement activation. CRP exhibits a good correlation with acute inflammation, and its levels reflect the inflammatory level to some extent [42–44]. We also observed neutrophil infiltration in the rat kidneys after glycerol treatment. The results showed that the expression of CD68 in the kidney increased significantly after glycerol injection. CD68 is considered the most widely used marker for macrophages. Macrophages, as critical immune effector cells, are derived from blood monocytes and can be divided into M1 (classically activated) and M2 (alternatively activated) subpopulations according to their functional phenotypes [45]. Enhanced CD68 expression reflects an increase in the number of macrophages in injured kidney tissues, and CVF could inhibit its expression. These results indicate that inflammation was involved in RM-induced AKI, and this inflammation was partially related to complement activation.

The expression of C5a in the kidney increased significantly after glycerol injection, and the highest expression occurred at 24 h. C5a is the product of C5 activation, and it exerts potent proinflammatory effects. C5a must combine with C5aR to exert its effects. C5aR is expressed in the kidney, primarily in renal mesangial cells and proximal and distal tubule epithelial cells. C5a is an anaphylatoxin and a potent chemotactic agent, and it promotes the adhesion and activation of leukocytes, including macrophages [46]. Neutralizing antibodies against C5a exhibited protective effects in experimental sepsis [47]. Treatment with a small molecule inhibitor of C5aR to block C5a signaling protected mice from injury in a renal I/R model [11]. Studies have shown that C5a can enhance the induction of macrophages and T cells indirectly through the production of IL-6 and TNF-α [48]. In our results, IL-6 mRNA and protein expression increased in the AKI group compared with that in the control group. The highest expression of CD68 and IL-6 occurred at 24 h, and CVF inhibited this increase, suggesting that these factors are relevant to complement activation. C5a is involved in the activation of monocytes, macrophages, and Th2 cells, resulting in an increase in IL-6 secretion. These cells interact and induce the production of more cytokines and inflammatory mediators and amplify the inflammatory response, causing renal tissue damage directly and indirectly in RM-induced AKI. C3a is also an anaphylatoxin, but it is not as potent a chemotactic agent as C5a. Mice with targeted genetic deletion of the C3a receptor are protected from AKI [49].

As mentioned above, complement-mediated inflammation is mainly due to the production of complement anaphylotoxins C5a and C3a. In addition, it has been reported that the membrane attack complex (MAC; C5b-9) induces macrophages to release IL-1β and IL-18 to promote the inflammatory immune response [50]. The MAC is the final product of complement activation, and it forms lytic pores in the outer membrane of target surfaces to lyse microorganisms and abnormal host cells. C5b-9 also activates cells in sublytic quantities. The proinflammatory consequences of sublytic MAC have been reported in many cell types [51,52]. Small molecule MAC blockers inhibit inflammation in various models [53]. One study showed that renal epithelial cells exposed to sublytic MAC released IL-6, IL-8, MCP-1, and VEGF [54]. Our data demonstrated that MAC expression in the kidney increased after glycerol injection, and CD68 expression also increased. Sublytic MAC may trigger macrophages to release of inflammatory cytokines, including IL-6, from host cells [52].

The MAC creates pores in the cell membrane, allowing Ca^2+^ to enter the cell, which leads to intracellular calcium overload, and exacerbating ATP depletion in hypoxic cells [55]. The ultimate result of these processes is apoptosis. Apoptosis is another important biological effect of complement. Many complement activation products, such as C5a, C1q, and CD59, affect apoptosis. Sepsis-associated apoptosis has been linked to a C5a-C5a receptor interaction, which leads to organ dysfunction, immunosuppression, and lethality [56]. C5a induced apoptosis in IR-induced acute lung injury by initiating Bcl-2 degradation [57]. Our study demonstrated that complement depletion down-regulated apoptosis in renal tissues of rats with RM. Our previous study showed that apoptosis was a prominent feature in RM-induced AKI [9]. The expression of complement components, including C5a, C1q, MAC, and CD59, which induce apoptosis, was enhanced in the present study. Apoptosis may be another important mechanism of complement in RM-induced AKI.

Complement activation is not unlimited, and complement regulatory proteins play important roles in preventing damage to normal cells caused by complement activation, with the role of cell-membrane-anchored proteins, such as CD55, CD59, CD46, and CD35, being particularly critical [58]. CD59 is attached to the cell surface by a glycosylphosphatidylinositol (GPI) anchor, which prevents C9 units from binding to the C5b-8 complex. Loss of CD59 increases the amount of C5b-9 complexes and leads to more functional transmembrane pores. CD59 knockout mice suggest that CD59 protects against ischemic brain damage [59]. The results of the present study demonstrate that CD59 expression in the renal tissues of normal rats increased gradually after intramuscular glycerol injections, but CVF significantly reduced this increase. These results indicate that increased CD59 expression was also related to complement activation. The early increase in CD59 expression in RM-induced AKI may inhibit MAC formation and reduce the inflammatory response and apoptosis, which increase initially as part of the body's own defense. This increase in CD59 expression was partially related to the stimulation of TNF-α, IL-1, and IL-6 [60], which increased passively. In conclusion, our results provide direct evidence that complement plays a critical role in RM-induced AKI in rats. Complement may be activated via multiple pathways, and activated complement exerts numerous biological effects that induce and/or aggravate kidney damage.

The longest observation time in this study was 72 h. Inhibition of complement can diminish the early inflammatory response and achieved a longer time benefit[61]. Over a longer observation time, damaged tissue may begin to repair, regenerate, or develop fibrosis, and whether complement continues to play a role requires further study. Most of the conclusions in this study were determined based on complement depletion via CVF injection prior to intramuscular injection of glycerol, which is not clinically feasible. To establish clinical applications, including post-treatment with CVF, further studies are required.

## Supporting information

S1 File The levels of renal function and apoptosis. (PDF)

S2 File The electrophoretogram, amplification curve and melt curve of Real-time qPCR. (PDF)
