# Supplementary material for: The role of complement activation in rhabdomyolysis-induced acute kidney injury
Source: PLoS One. 2018 Feb 21;13(2):e0192361. doi: 10.1371/journal.pone.0192361 (PMC5821337; doi:10.1371/journal.pone.0192361)
Supplement: S1 File — (PDF) [file pone.0192361.s001.pdf]

Table A Levels of Serum BUN and Cr in the Two Groups (means±SD, n = 6).

Group	Time	BUN (mg/dl)	Cr (mg/dl)
Control	2 h	15.68±1.44	0.28±0.01
	6 h	16.28±1.54	0.25±0.02
	24 h	16.74±1.82	0.30±0.02
	72 h	14.87±1.68	0.27±0.02
cvf	2 h	16.46±1.37●	0.27±0.01●
	6 h	15.43±1.47●	0.31±0.02●
	24 h	17.36±1.84●	0.28±0.01●
	72 h	15.88±1.57●	0.28±0.02●

● P>0.05 vs. control

Table B Apoptosis rate of each group(means±SD, n = 6)

Group	2 h	6 h	24 h	72 h
Control	0	0	0	0
AKI	4.45±1.82*	29.71±3.46*	50.27±6.35*	34.23±4.17*
CVF+AKI	4.18±1.67*●	22.48±2.35*#	34.67±5.19*#	28.46±4.63*#

* P<0.01 vs. control; ● P>0.05, # P<0.01 vs. AKI
