# Supplementary material for: The role of complement activation in rhabdomyolysis-induced acute kidney injury
Source: PLoS One. 2018 Feb 21;13(2):e0192361. doi: 10.1371/journal.pone.0192361 (PMC5821337; doi:10.1371/journal.pone.0192361)
Supplement: S2 File — (PDF) [file pone.0192361.s002.pdf]

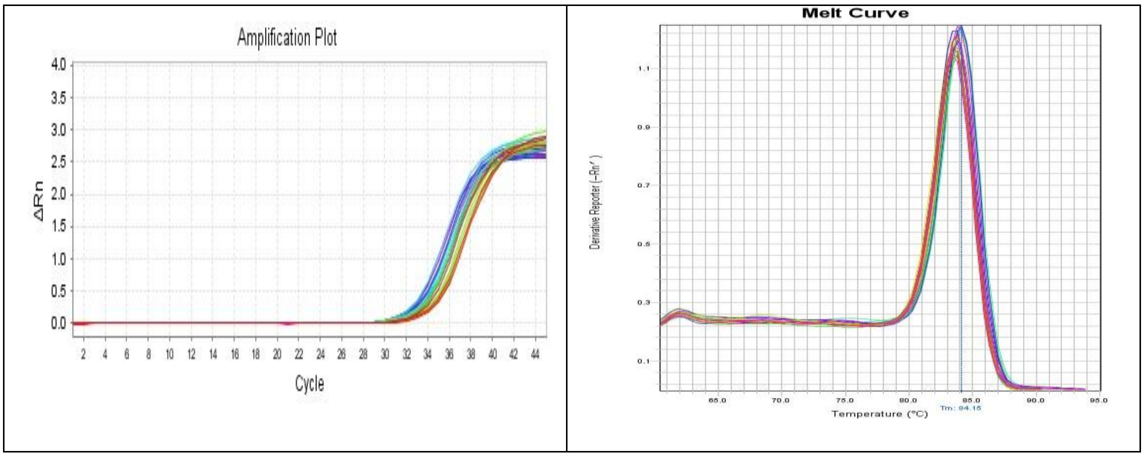

Figure A **The amplification curve and melt curve of C1q mRNA**

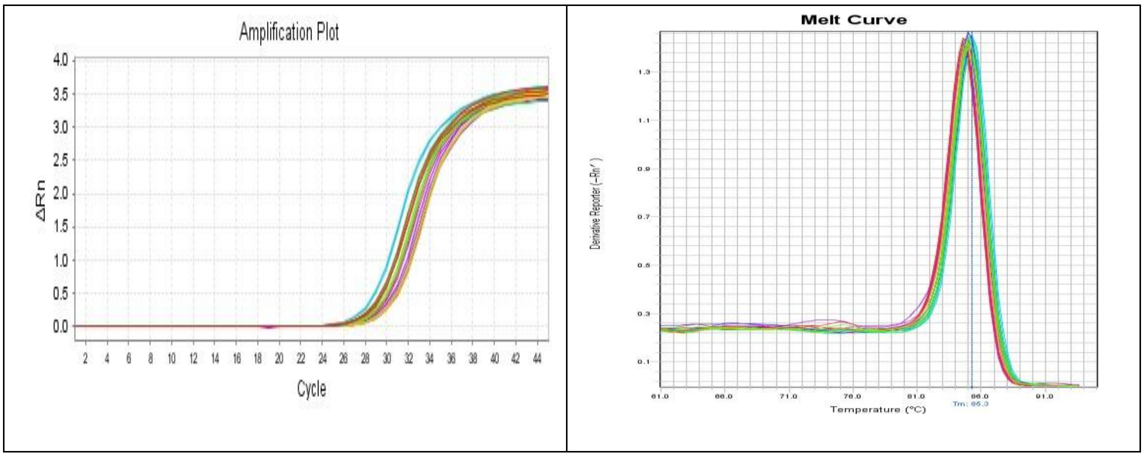

Figure B **The amplification curve and melt curve of fB mRNA**

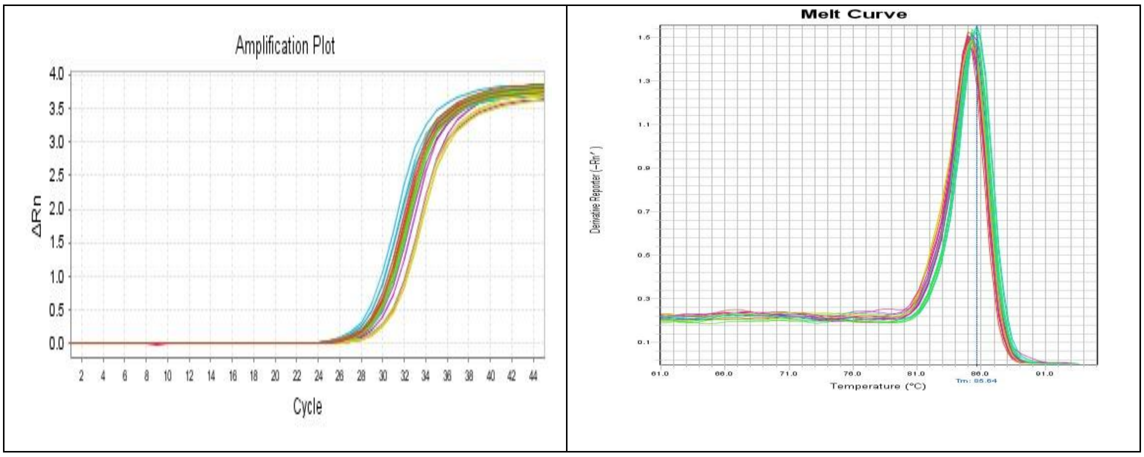

Figure C **The amplification curve and melt curve of MBL-A mRNA**

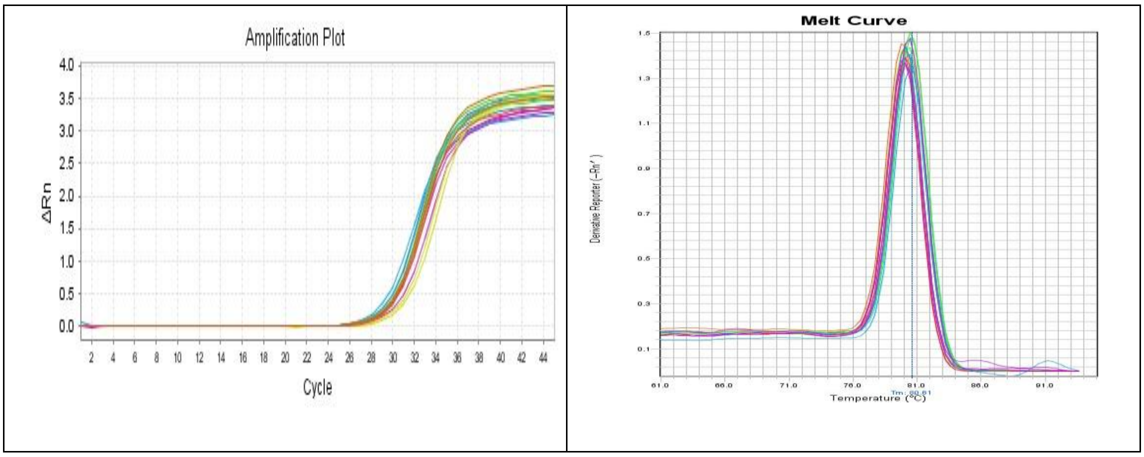

Figure D **The amplification curve and melt curve of IL-6 mRNA**

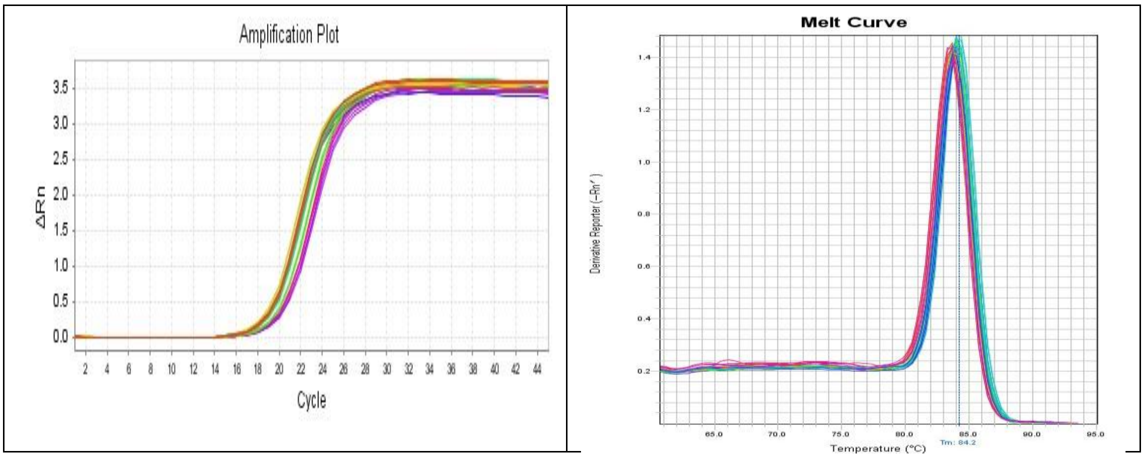

Figure E **The amplification curve and melt curve of GAPDH**
